# Three‐year myopia management efficacy of extended depth of focus soft contact lenses (MYLO) in Caucasian children

**DOI:** 10.1111/opo.13549

**Published:** 2025-06-30

**Authors:** Sergio Díaz‐Gómez, Padmaja Sankaridurg, José A. López‐Garrido, Mercedes Burgos‐Martínez, Jesús Carballo‐Álvarez

**Affiliations:** ^1^ Faculty of Optics and Optometry Complutense University of Madrid Madrid Spain; ^2^ Miranza Centro Oftalmológico Integral (COI) Bilbao Spain; ^3^ School of Optometry and Vision Science University of New South Wales Sydney New South Wales Australia; ^4^ Mark'ennovy Madrid Spain

**Keywords:** Caucasian, contact lenses, cumulative absolute reduction in axial elongation, extended depth of focus, myopia

## Abstract

**Purpose:**

To evaluate the progression of myopia as assessed by changes in axial length (AL) and spherical equivalent (SE) in Caucasian children wearing extended depth of focus soft contact lenses (CL) compared with distance single‐vision spectacles after 3 years.

**Methods:**

A total of 96 progressing (at least −0.75 D increase in the previous 12 months) myopic children (aged 6–13 years) with SE ranging from −0.75 to −10.00 D were recruited. Forty‐five children were fitted with CL (MYLO), whereas 51 wore spectacles. Cycloplegic refraction was measured with an auto‐refractometer and AL with the Zeiss IOLMaster‐700 at 6‐month intervals. Subjective responses of CL wear were determined using a questionnaire on a scale of 1 (very poor) to 10 (excellent). High‐contrast visual acuity (HCVA) was also evaluated.

**Results:**

A total of 43 and 41 children achieved 3 years of wear with CL and spectacles, respectively. Mean change in SE and AL was −0.90 ± 0.36 D and 0.55 ± 0.04 mm in the CL group and −1.64 ± 0.26 D and 0.97 ± 0.03 mm in the spectacle group, respectively (all *p* < 0.001). The cumulative absolute reduction in axial elongation was 0.42 mm. The difference in SE change was 0.74 D. While 90% of the children wearing CL had an AL increase of ≤0.60 mm, 96% of the spectacle group increased by at least 0.91 mm. Indeed, 50% of the CL group showed myopia progression of at least 0.75 D, whereas all the spectacle group progressed by at least 0.75 D. Subjective responses indicated a mean value ≥9. There was a reduction in HCVA of half a logMAR line with CL compared to spectacles (*p* < 0.001).

**Conclusions:**

Three years of wear with MYLO CL resulted in clinically significant slower myopia progression compared to the SV spectacle group.

**Key points**

- This study highlights the myopia management efficacy of soft extended depth of focus contact lenses in Caucasian children.
- Three years of contact lens wear resulted in clinically significant slower myopia progression compared to single‐vision spectacles.
- Contact lens wear provided good vision and was safe over the whole 3‐year follow‐up period.

## INTRODUCTION

It is estimated that by 2050, nearly half of the world population will be myopic, leading to a concomitant increase in ocular complications related to excessive axial elongation.^1^ Myopia prevalence begins to increase noticeably after 6 years of age, and tends to stabilise in late adolescence.^2^ In East and Southeast Asia, the prevalence of myopia in young adults is approximately 80%–90%.^3^, ^4^ A recent study of Spanish children aged 5–7 years found that the prevalence of myopia was 19% from 2016 to 2021, with projections indicating an increase to 30.2% by 2030.^5^ Therefore, it is necessary to implement interventions to slow the progression of myopia among children and adolescents.^6^

Currently, several options exist to slow myopia progression, both as individual or combination treatments.^7^, ^8^ These include spectacles with specific designs,^9^, ^10^ pharmacological treatments with low‐dose concentrations,^11^ low‐level red‐light therapy,^12^ contact lenses (CL), including orthokeratology with rigid gas permeable CL^13^ and soft CL designed for myopia management.^14^ Moreover, there is consensus on the protective effect of time spent outdoors on the onset of myopia or other environmental interventions to modify the performance of near work and promote healthier lifestyles in children.^15^, ^16^

It is well established that optical defocus regulates eye growth, but little is known about the specific signal and the pathway.^17^, ^18^ Most soft CL designed for myopia control include a zone or region that corrects the distance refractive error as well as areas with varied power to induce peripheral myopic defocus or simultaneous competitive defocus, thus reducing myopia progression.^19^ Utilising this approach, multiple power profile CL have been developed, including extended depth of focus (EDOF),^20^, ^21^ multi‐concentric with annular rings,^14^, ^22^ centre‐distance multifocal,^23^ non‐coaxial toroidal^24^ and an asymmetrical power map.^25^ Specifically, the EDOF soft CL incorporates higher order aberrations that aim to reduce the image quality for points posterior to the peripheral retina. A recent study reported that pupil size may modulate the effectiveness of myopia control, so that EDOF lenses might be more effective for subjects with pupil sizes <3.00 mm than other soft CL designs.^26^

The purpose of the current study was to evaluate the rate of myopia progression over 3 years, as assessed by the change in axial length (AL) and cycloplegic refraction from baseline, in progressing Caucasian myopic children using EDOF soft CL versus single‐vision distance spectacles (SV‐spectacles). Two‐year follow‐up results were published previously.^27^

## METHODS

### Study Design and Participants

A total of 96 myopic children between 6 and 13 years old were recruited in this prospective, non‐randomised, comparative clinical trial conducted at Clínica de Oftalmología Integral in Bilbao, Spain. All participants were of European (Caucasian) ethnicity, had normal ocular health that did not preclude CL wear, myopia progression of at least 0.75 D in the previous 12 months as determined by cycloplegic refraction, best corrected visual acuity of 0.00 logMAR or better in both eyes, cycloplegic spherical equivalent (SE) ranging from −0.75 to −10.00 D and astigmatism ≤0.75 D. Children with a history of systemic or ocular disease and current use of systemic or topical ocular medications were excluded. Subjects with prior CL wear or any myopia control treatment were also excluded to avoid potential confounding effects related to previous fitting or potential biases in their subjective responses.

Table 1 lists the results of previous studies using EDOF CL with either the same or similar designs to that used in the current study.^20^, ^21^, ^27^ Table 2 presents the findings of previous 3‐year investigations of soft CL for myopia management.^23^, ^28^, ^29^ Since the efficacy of the EDOF lens for myopia control has been reported previously, a randomised study was not considered here for ethical reasons. In the current trial, children were allocated either to distance single‐vision (SV) spectacles if they did not want to wear myopia management CL, rather than being randomised, as the motivation and willingness of children and parents are important both for retention in the trial and success in myopia management.^30^ Therefore, those interested in taking part in the study and being fitted with soft CL were screened for suitability, and assigned to the CL group if they met the inclusion criteria. CL and maintenance solutions were provided at no charge. If subjects were interested in participating in the trial but not interested in CL wear, they were assigned to the spectacle group to wear their SV distance spectacles (not provided in the trial). When the study began, spectacle lenses designed for myopia control were not commercially available. Therefore, experimental group participants wore SV distance spectacles when not wearing their CL. A total of 45 participants (two replaced at the first visit) were assigned to wear EDOF CL, while 51 participants were assigned to the spectacle control group.

**TABLE 1:** Previous publications about myopia management with similar EDOF contact lens (CL) designs.

Authors	Study design		AL mean changes (mm)			SE mean changes (D)		
			Control group	CL group	CARE	Control group	CL group	Difference
Manoharan et al.^21^	EDOF CL (*n* = 31) versus SV‐spectacle (*n* = 38)	1‐year	0.22 ± 0.03	0.11 ± 0.03	0.11	−0.48 ± 0.07	−0.20 ± 0.08	0.28
	Asian (India)							
	SE: −0.50 to −5.75 D							
	Age: 7–15 years							
Diaz‐Gómez et al.^27^	EDOF CL (*n* = 45) versus SV‐spectacle (*n* = 45)	First‐year	0.34	0.19	0.15	−0.57	−0.34	0.23
	Caucasian (Spain). Progressors	Second‐year	0.32	0.18	0.14	−0.56	−0.28	0.28
	SE: −0.50 to −10.00 D	Whole period	0.66 ± 0.03	0.37 ± 0.04	0.29	−1.13 ± 0.20	−0.62 ± 0.30	0.51
	Age: 6–13 years							
Sankaridurg et al.^20^	EDOF CL (98) versus SV‐CL (*n* = 102)	First‐year	0.33	0.22	0.11	−0.66	−0.47	0.19
	Asian (China)	Second‐year	0.25	0.23	0.02	−0.46	−0.34	0.12
	SE: −0.75 to −3.50 D	Whole period	0.58 ± 0.27	0.45 ± 0.28	0.13	−1.12 ± 0.51	−0.81 ± 0.65	0.31
	Age: 7–13 years							

*Note*: AL and SE changes expressed in mean or mean ± SD.

Abbreviations: AL, axial length; CARE, cumulative absolute reduction in axial elongation; EDOF, extended depth of focus; SE, spherical equivalent; SV, single vision.

**TABLE 2:** Previous publications regarding myopia management with soft contact lenses (CL) in a 3‐year follow‐up period.

Authors	Study design	Follow‐up	AL mean changes (mm)			SE mean changes (D)		
			Control group	CL group	CARE	Control group	CL group	Difference
Ruiz‐Pomeda et al.^28^	Dual‐Focus CL (*n* = 13) versus SV‐spectacles (*n* = 24)	First year	0.23	0.09	0.14	−0.41	−0.17	0.24
Mass study	Nearly 100% Caucasians (Spain)	Second year	0.19	0.14	0.05	−0.26	−0.24	0.04
	SE: −0.75 to −4.00 D	Third year	0.21	0.16	0.05	−0.55	−0.37	0.18
	Age: 8–12 years	Whole period	0.63	0.39	0.24	−1.22	−0.78	0.44
Walline et al.^23^	Multifocal CL Add +1.50 D (*n* = 98) versus SV‐CL (*n* = 98)	First year	0.29	0.26	0.03	−0.43	−0.35	0.08
BLINK study	Multi‐Race, Mostly Caucasian (USA)	Second year	0.21	0.18	0.03	−0.31	−0.30	0.01
	SE: −0.75 to −5.00 D	Third year	0.16	0.14	0.02	−0.31	−0.24	0.07
	Age: 7–11 years	Whole period	0.66	0.58	0.08	−1.05	−0.85	0.16
Walline et al.^23^	Multifocal CL Add +2.50 D (*n* = 98) versus SV‐CL (*n* = 98)	First year	0.29	0.16	0.13	−0.43	−0.20	0.23
BLINK study	Multi‐Race, Mostly Caucasian (USA)	Second year	0.21	0.14	0.07	−0.31	−0.21	0.10
	SE: −0.75 to −5.00 D	Third year	0.16	0.12	0.04	−0.31	−0.19	0.12
	Age: 7–11 years	Whole period	0.66	0.42	0.24	−1.05	−0.60	0.45
Chamberlain et al.^29^	Dual‐Focus CL (*n* = 53) versus SV‐CL (*n* = 56)	First year	0.24	0.09	0.15	−0.58	−0.18	0.40
	Multi‐Race, nearly 50% white (Portugal, UK, Singapore and Canada)	Second year	0.21	0.12	0.09	−0.34	−0.20	0.14
	SE: −0.75 to −4.00 D	Third year	0.17	0.09	0.08	−0.32	−0.13	0.19
	Age: 8–12 years	Whole period	0.62	0.30	0.32	−1.24	−0.51	0.73

Abbreviations: AL, axial length; BLINK, Bifocal lenses in near‐sighted kids; CARE, cumulative absolute reduction in axial elongation; SE, spherical equivalent; SV, single vision.

### Contact lenses

As described previously, the MYLO® lens (mark'ennovy, markennovy.com) is a soft silicone hydrogel CL (Filcon 5B (60) [75%]) featuring an EDOF design that incorporates a refractive, non‐monotonic and aperiodic power profile that results in an EDOF equivalent to 1.50 D (total range of the extended depth of focus). The lens is available as daily wear and monthly replacement lenses customised for individual ocular parameters. Lens parameters were calculated considering the topographic anterior corneal values (i.e., keratometry, horizontal visible iris diameter and corneal eccentricity) and the cycloplegic refraction referenced to the corneal plane. Fifteen minutes after lens fitting, a slit‐lamp evaluation (Topcon SL‐D4, topconhealthcare.com) was performed to check that the lens had acceptable centration and movement. Participants were instructed to wear the CL for at least 10 h per day, 6 days a week, during all waking hours. Wearing time was recorded using a take‐home questionnaire. Lenses and lens cases were replaced monthly. Alvera (Avizor, avizor.com), a multipurpose solution, was provided to the CL wearers for lens maintenance. The composition of the solution included aloe vera, poloxamer, polyvinylpyrrolidone (PVP), disodium edetate (EDTA) and polyhexanide (PHMB) 0.0002%. In the control group, children were encouraged to wear their spectacles during all waking hours every day.

### Subjective measurements

Subjective responses related to the vision and comfort of the CL were determined using a questionnaire where responses were ranked from 1 (very poor) to 10 (excellent) in 1‐unit steps as described previously.^24^, ^31^, ^32^, ^33^ Questions asked about handling comfort, clarity of vision, vision stability, overall satisfaction and willingness to continue wearing the lens. The questionnaire was administered after 1 month and 3 years of lens wear.

### Ocular and visual outcomes

All trial‐related measurements were conducted by a single optometrist (S. D‐G.) at baseline and 6‐monthly intervals. Two ophthalmologists evaluated the ocular health of the participants. Slit‐lamp examination included assessment of the cornea, conjunctiva, eyelids and lid margins of both eyes. Corneal and conjunctival integrity was confirmed with a fluorescein examination. Corneal diameter, power, radius of curvature (CR) and eccentricity were assessed with the Topographer‐aberrometer OPD Scan III (Nidek, nidek.com). The IOLMaster® 700 Biometer (Carl Zeiss Meditec AG, zeiss.com) was used to obtain AL prior to cycloplegia. Two drops of 1% cyclopentolate (Alcon‐Cusí, alcon.com) were instilled into each eye of the participants, 5 min apart. Twenty minutes after instillation of the second drop, three measurements of the cycloplegic refractive error were obtained with an auto‐refractometer (Topcon‐TRK‐2P, topconhealthcare.com) and the mean SE calculated. The AL/CR ratio was quantified. The efficacy of myopic control of the CL was determined by evaluating the difference in AL progression between the test and control groups and defined as the Cumulative Absolute Reduction in Axial Elongation (CARE).^34^ Similarly, the difference in SE progression between the experimental and control groups was also computed. Visual acuity was measured using the OptoTab (smarthings4vision.com) at 5 m under photopic conditions (≥85 cd/m^2^) either with the spectacle correction or CL. Subjects were encouraged to guess the identity of the letters if unsure. At each visit, a spherical over‐refraction was performed monocularly for each eye to determine the power that yielded the best‐corrected visual acuity.

### Statistical analysis

All statistical tests were performed using SPSS version 28.0 (ibm.com). Descriptive statistics, including the mean and standard deviations of the quantitative data, were calculated. Changes in AL and SE were the primary outcome variables. The normality of all data was checked using the Kolmogorov–Smirnov test. The Mann–Whitney U test was used to compare the outcomes at each visit between the spectacle and CL groups. Comparisons between the outcomes obtained at baseline and every subsequent 6 months were carried out using the Friedman test followed by Bonferroni's post hoc test. Comparisons of AL, SE and high contrast visual acuity (HCVA) values in the follow‐up periods between the spectacle and CL groups were performed using a repeated measures ANOVA with a Greenhouse–Geisser correction followed by Bonferroni's post hoc test. Spearman's correlation coefficient was used to analyse the relationship between the baseline outcomes and the change in AL or SE. The relationship between the changes in AL and SE over the 36‐month period was also examined. The Greenhouse–Geisser test was used to analyse the within‐subjects effects related to confounders (right/left eyes, sex and myopic parents). Statistical significance was set at *p* < 0.05 (*p < 0.05; **p < 0.01; ***p < 0.001).

## RESULTS

**FIGURE 1:**
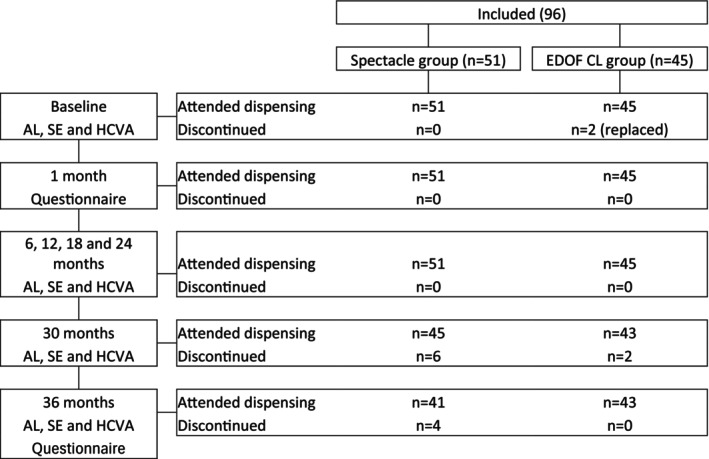
Flow chart of the participants that attended, were replaced or discontinued at each visit. Realised measurements: AL, axial length; CL, contact lens; EDOF, extended depth of focus; HCVA, high contrast visual acuity; SE, spherical equivalent.

Figure 1 shows the progression of children through the trial. Two subjects dropped out of the CL group at the start due to handling related issues and were replaced by another two participants. After 24 months, six participants who wore spectacles requested to move into the CL group due to rapid progression in myopia. They were provided with test CL but discontinued from the study. At the 30‐month visit, two participants from the test group discontinued due to retinal pathology (posterior staphyloma, not related to contact lens wear) and change of residence, respectively. Their data were removed to avoid bias. An additional four spectacle wearers also dropped out of the study. Two were due to an increase in myopia, and the participants switched to myopia management options (one to orthokeratology and the other to myopia control spectacles, while the remaining two relocated to another city).

**FIGURE 2:**
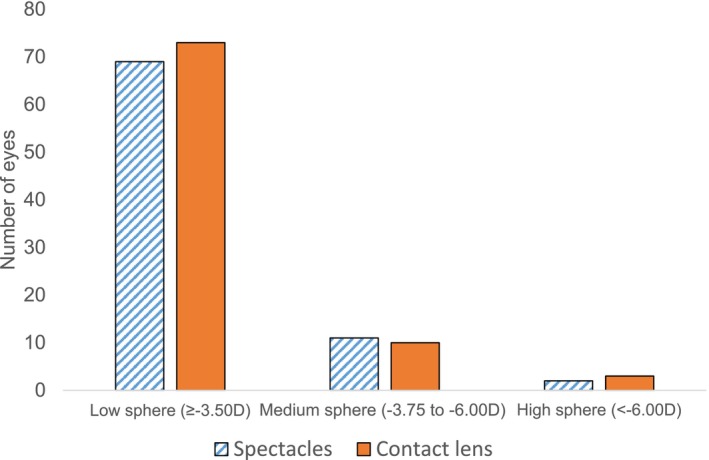
Number of eyes with low, medium and high myopia in the spectacle and contact lens groups.

**FIGURE 3:**
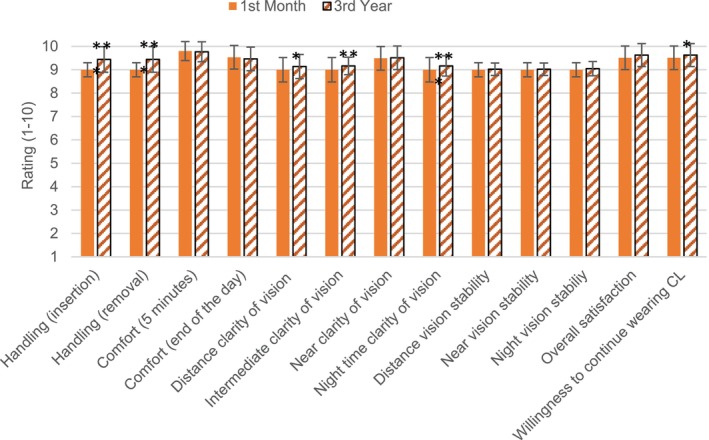
Subjective responses after 1 month and 3 years of contact lens (CL) wear. Results are shown as mean ± SD (error bars). *p < 0.05; **p < 0.01.

A total of 43 and 41 participants wearing MYLO CL and control spectacles, respectively, completed the 36‐month follow‐up visit. All data from children who were discontinued from the study were discarded. Table 3 presents the baseline demographic data of the 84 participants who completed the 3‐year study. While the mean baseline AL was 1 mm greater in the spectacle group (*p* < 0.001), there were no significant differences between the groups for SE (*p* = 0.16). However, the spectacle lens group also had a higher baseline AL/CR ratio (*p* < 0.001). As shown in Figure 2, there was a similar distribution of myopia across the two groups (low myopia (≥−3.50 D): 85% vs 84%, medium (−3.75 to −6.00 D) myopia: 12% vs 13% and high myopia (<−6.00 D): 3% vs 2%, for the CL and control groups, respectively). Questionnaire responses after wearing the CLs are represented in Figure 3. All items showed a mean rating value of 9 or higher. Some non‐clinically significant improvements were found after 3 years compared with the 1‐month values.

**TABLE 3:** Demographic outcomes in the spectacle and contact lens groups.

	Spectacles	Contact lens	*p*‐Value
*n*	41	43	
Age (years/range)	11.1 ± 1.2 (6–13)	10.8 ± 1.2 (7–13)	0.06
Male/female	17/24	19/24	
No myopic parents	12 (30%)	6 (14%)	
One myopic parent	19 (46%)	30 (70%)	
Both parents myopic	10 (24%)	7 (16%)	
AL (mm)	25.55 ± 0.87	24.54 ± 0.94	<0.001
SE (D)	−2.68 ± 1.01	−2.61 ± 1.55	0.16
KM (D)	41.12 ± 1.14	42.16 ± 0.99	<0.001
CR (mm)	8.21 ± 0.22	8.01 ± 0.19	<0.001
AL/CR	3.11 ± 0.03	3.06 ± 0.06	<0.001

*Note*: Age, AL and SE expressed as Mean ± SD.

Abbreviations: AL, axial length; AL/CR, AL (mm) divided by CR (mm); CR, mean corneal radius of curvature; KM, mean corneal power; SE, spherical equivalent.

In the CL group, there were 31 cases of mild corneal staining, 15 of which appeared in the last year but did not necessitate discontinuation of lens wear. Some incidents of torn or broken lenses, especially at the beginning of the trial, were considered acceptable.

**FIGURE 4:**
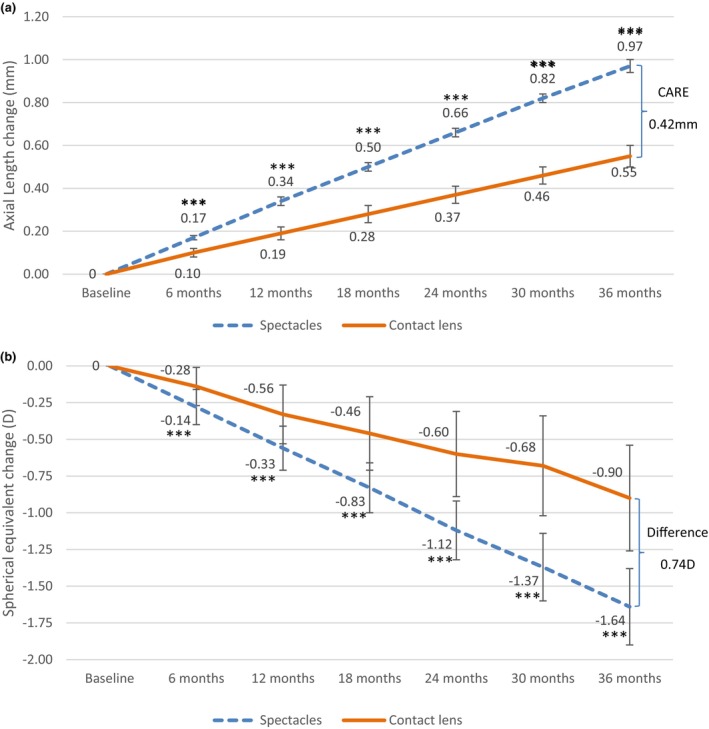
Mean change in axial length (a) and spherical equivalent refractive error (b) in the spectacle and contact lens groups for the 36‐month study period. Error bars represent SD. ***p < 0.001. CARE, Cumulative Absolute Reduction in Axial Elongation.

The AL and SE results at each visit are presented in Tables 4 and 5. For each group, Bonferroni's post hoc test indicated statistically significant differences in AL and SE changes between all of the measured times (*p* < 0.001). Figure 4 illustrates the mean changes (and standard deviations) in AL and SE over the follow‐up period. Over 3 years, significantly less axial elongation and change in SE was observed in the CL group, compared with the spectacle group (*p* < 0.001). The increase in AL over the 3‐year period was 0.55 ± 0.05 mm (range: 0.44–0.66) in the CL group and 0.97 ± 0.03 mm (range: 0.88–1.03) in the spectacle group, resulting in a CARE value of 0.42 mm. The total change in SE for the CL and spectacle group was −0.90 ± 0.36 D (range: −0.25 to −1.75 D) and −1.64 ± 0.26 D (range: −1.00 to −2.75), respectively. The mean difference in SE change between the two groups was −0.74 D.

**TABLE 4:** Axial length and spherical equivalent in the spectacle and contact lens groups measured at baseline. 6‐, 12‐, 18‐, 24‐, 30‐ and 36 months (M).

	Baseline	6 M	12 M	18 M	24 M	30 M	36 M	*p*‐Value
*Spectacles*								
AL (mm)	25.55 ± 0.87	25.71 ± 0.87	25.88 ± 0.87	26.05 ± 0.87	26.21 ± 0.87	26.36 ± 0.87	26.51 ± 0.87	<0.001
SE (D)	−2.68 ± 1.02	−2.97 ± 1.04	−3.24 ± 1.06	−3.51 ± 1.07	−3.79 ± 1.09	−4.06 ± 1.13	−4.32 ± 1.14	<0.001
*Contact lens*								
AL (mm)	24.54 ± 0.94	24.64 ± 0.94	24.73 ± 0.94	24.81 ± 0.94	24.91 ± 0.95	25.00 ± 0.95	25.09 ± 0.95	<0.001
SE (D)	−2.61 ± 1.55	−2.74 ± 1.56	−2.93 ± 1.64	−3.06 ± 1.68	−3.21 ± 1.67	−3.29 ± 1.70	−3.51 ± 1.70	<0.001

*Note*: AL and SE are expressed in Mean ± SD. Statistically significant difference at *p* < 0.05.

Abbreviations: AL, axial length; M, months; SE, spherical equivalent.

**TABLE 5:** Adjusted mean with 95% CI (lower; upper).

	Baseline	6 M	12 M	18 M	24 M	30 M	36 M
*Spectacles*							
AL (mm)	25.55 (25.36; 25.74)	25.72 (25.53; 25.91)	25.89 (25.70; 26.08)	26.05 (25.86; 26.24)	26.21 (26.02; 26.40)	26.36 (26.17; 26.55)	26.52 (26.32; 26.71)
SE (D)	−2.68 (−2.90; −2.55)	−2.97 (−3.20; −2.74)	−3.24 (−3.47; −3.01)	−3.51 (−3.75; −3.28)	−3.80 (−4.04; −3.56)	−4.06 (−4.31; −3.81)	−4.32 (−4.57; −4.07)
*Contact lens*							
AL (mm)	24.54 (24.34; 24.75)	24.65 (24.44; 24.85)	24.74 (24.53; 24.94)	24.83 (24.62; 25.03)	24.91 (24.71; 25.12)	25.00 (24.80; 25.21)	25.10 (24.89; 25.30)
SE (D)	−2.68 (−2.94; −2.27)	−2.74 (−3.08; −2.49)	−2.93 (−3.28; −2.58)	−3.06 (−3.42; −2.71)	−3.21 (−3.57; −2.85)	−3.29 (−3.66; −2.93)	−3.51 (−3.87; −3.14)

Abbreviations: AL, axial length; CI, confidence intervals; SE, spherical equivalent.

**FIGURE 5:**
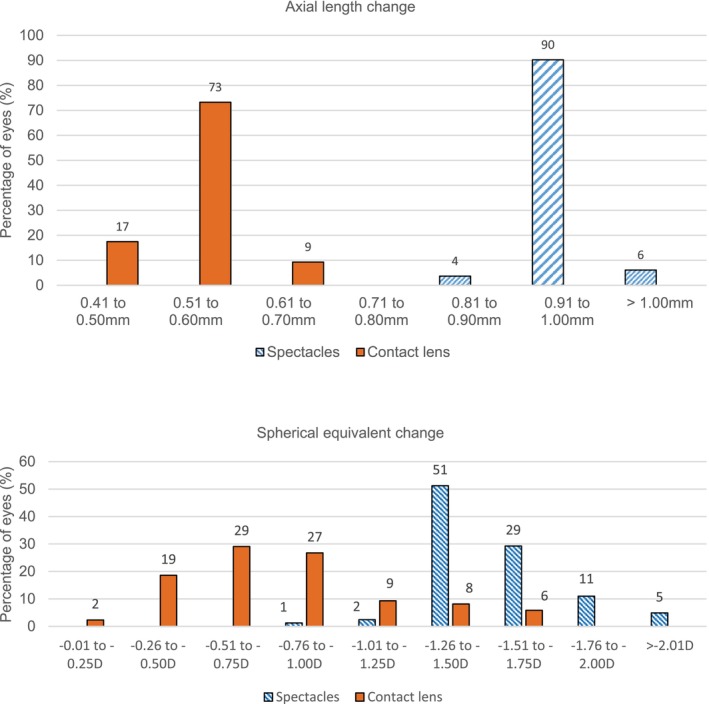
Distribution of changes in axial length and spherical equivalent in the spectacle and contact lens groups during the 36‐month study period.

Figure 5 illustrates the distribution of the change in AL and SE. Ninety per cent of the children wearing CL had an AL increase of 0.60 mm or less, while 96% of participants in the spectacle group increased by at least 0.91 mm. Additionally, 50% of the CL group showed a progression in SE of no more than 0.75 D. In contrast, all participants of the spectacle group developed at least 0.75 D more myopia.

When the relationship between the baseline values, AL change and SE change over the whole period was analysed, no significant correlation was found between the AL and SE changes for either group. Only a weak correlation was observed between baseline SE and the change in SE for the spectacle (rho 0.22/*p* = 0.04) and CL (rho 0.23/*p* = 0.03) groups. Those with a more myopic baseline SE showed a larger change in SE at the end of the 3‐year follow‐up. There were no significant differences in AL or SE progression between the right and left eyes for both control and CL groups. There were also no significant differences based on sex, number of myopic parents or their combined effects (Greenhouse–Geisser test).

Monocular (right eye) and binocular HCVA at baseline, 12, 24 and 36 months are presented in Table 6. Over the study period, the VA in the CL group was worse, compared with the spectacle wearers. However, the difference was only around half a line (2–3 logMAR letters) for both monocular and binocular HCVA (*p* < 0.001).

**TABLE 6:** Monocular and binocular photopic visual acuity in the spectacle and contact lens groups measured at baseline and 12, 24 and 36 months (M).

		Baseline RE	12 M RE	24 M RE	36 M RE	Baseline BE	12 M BE	24 M BE	36 M BE
HCVA (logMAR)	Spectacles	−0.03 ± 0.04	−0.03 ± 0.04	−0.03 ± 0.04	−0.03 ± 0.03	−0.04 ± 0.04	−0.04 ± 0.04	−0.04 ± 0.04	−0.04 ± 0.04
	Contact lens	0.04 ± 0.02	0.02 ± 0.02	0.02 ± 0.02	0.02 ± 0.02	0.03 ± 0.02	0.01 ± 0.02	0.01 ± 0.02	0.01 ± 0.02
	*p*‐value	<0.001	<0.001	<0.001	<0.001	<0.001	<0.001	<0.001	<0.001

*Note*: Data are expressed as mean ± SD except for *p*‐values. Statistically significant difference at *p* < 0.05.

Abbreviations: BE, both eyes; HCVA, high contrast visual acuity; RE, right eye.

## DISCUSSION

To the best of our knowledge, this is the first trial to report the 3‐year myopia control efficacy of an EDOF optical design CL in an entirely Caucasian group of children. Hence, it was expected that progression, with respect to the change in AL and SE, would be slower than that observed in an Asian population.^20^ However, it should also be noted that the study included only those participants whose myopia had progressed by at least 0.75 D over the 12 months prior to enrolment, and therefore, these were fast progressors. The efficacy of myopia control soft CL in this group is not well understood. Aller et al. fitted bifocal soft CL (Acuvue, acuvue.com) to a group of multi‐ethnic children who progressed by 0.50 D or more prior to their visit, and found that AL increased slowly at only 0.05 mm over a 1‐year follow‐up. In contrast, the control group, fitted with SV‐CL, increased by 0.24 mm, resulting in a CARE value of 0.19 mm that was higher than that found in the present study (0.15 mm).^35^ The present findings agree with those reported in a retrospective cohort analysis by Cooper et al. (CARE of 0.45 mm at 3 years) using the EDOF NaturalVue multifocal lens (vtivision.com) fitted in subjects who showed ≥0.50 D of myopic progression in at least one eye prior to wearing the lens.^36^

Importantly, the mean difference of 0.74 D between the test and control groups was significant and very close to the requirements set by the US Food and Drug Administration (FDA) guidelines for a minimum difference in refractive error of 0.75 D between the experimental and control groups over a period of 3 years.^37^

Although the groups were not randomised, they shared similar baseline characteristics, apart from the baseline AL and AL/CR. However, the baseline AL and CR did not correlate significantly with the AL changes over time, suggesting that this difference may not have been crucial to the observed outcome.

Previous studies with Asian children tested the same or similar EDOF lens designs on a daily replacement basis (Table 1). Notably, a recent study in an Indian population with a lens having the same optical profile and a SV‐spectacle control group observed a CARE value of 0.11 mm and a SE difference of 0.28 D after 1 year of wear; similar to the present study results of 0.15 mm and 0.23 D, respectively.^21^ Sankaridurg et al. evaluated an EDOF CL (Aquamax test III, pegavision.com) over a 2‐year period with Chinese children, using SV CL as a control. The results for their control group are in agreement (AL and SE change of 0.58 mm and −1.12 D, respectively) with the SV‐spectacle group of the current study (0.66 mm and −1.12 D). However, they found a higher increase in AL and SE (0.45 mm and −0.81 D, respectively) in the EDOF CL group than the current study (0.37 mm and −0.60 D).^20^ This may be related to differences between the studies in terms of race, range of refractive errors (from –0.75 to −3.50 D vs −0.75 to −10.00 D), loss to follow‐up (these authors reported that 25.4% of children discontinued prior to the 1‐month study visit, and at least 38% did not complete the 2‐year follow‐up), wearing time of the CL (at least 8 h per day compared with at least 10 h) and all the subjects in the present investigation being fast progressors.

Unlike previous trials with soft contact lenses for myopia control, the current study included a wider range of SEs and higher mean AL and SE. Tideman et al. indicated that a longer AL may be a predictor of greater myopia progression.^2^ The current AL change data over 3 years indicated that these eyes were probably in the higher percentiles for AL.^2^ Furthermore, although the baseline SE was similar between the test and control groups, the baseline AL/CR was higher in the spectacle group, indicating that these eyes were probably at greater risk of progression. This may explain why the 3‐year axial elongation (0.97 mm) and myopia progression (−1.64 D) seen in the spectacle group was higher than previously reported over the same duration of follow‐up (Table 2). Ruiz‐Pomeda et al. reported a mean progression of 0.63 mm and −1.22 D in the SV‐spectacle group of the Misight Assessment Study Spain (Mass) with Caucasian children.^28^ Furthermore, Chamberlain et al. noted a mean AL and SE change of 0.62 mm and −1.24 D, respectively, in children wearing SV CL and having the same baseline age and SE as Ruiz‐Pomeda et al.^29^ Walline et al., in a clinical trial of children younger than those in the present study (7–11 years of age) wearing SV CL, showed a mean progression of 0.62 mm and −1.01 D.^23^ Slightly larger values were found by Zadnik et al. in the placebo group of the CHAMP study (0.81 mm and −1.28 D), with participants between 3 and 17 years of age and a SE ranging from −0.50 D to −6.00 D.^38^ Likewise, the results of the COMET study conducted by Gwiazda et al. showed a mean increase of 0.75 mm and −1.48 D in the SV‐spectacle group with 264 children aged from 6 to 11 years having a mean baseline SE of −2.37 ± 0.84 D.^39^ Interestingly, AL progression in the spectacle group of the current study was similar to that reported by Hiraoka et al. (1.00 mm) in a control group of 30 Japanese subjects who preferred to use distance spectacles rather than undergo orthokeratology, aged 8–12 years (present study 8–13 years), with SE from −0.75 D to 4.63 D at baseline.^30^ Further, the AL progression in the current spectacle group was similar to that found in the control group of Lam et al., who showed an increase of 0.92 mm. These authors analysed the efficacy of Defocus Incorporated Multiple Segments (DIMS) spectacle lenses in a sample of 55 Chinese children with a mean AL of 24.57 mm, SE ranging from −1.00 D to −5.50 D and ages from 10 to 15 years.^10^

In terms of AL change, the myopic changes observed in the present study were similar over the entire follow‐up period in both the experimental and control groups. In agreement, Sankaridurg et al. found a similar AL change when using the same EDOF CL design (test III) as the current study between the first and the second year (0.22 and 0.23 mm, respectively) in Asian children. However, unlike the present investigation, their CARE value decreased from 0.11 m in the first year to only 0.02 mm in the second year. This was due to the reduction in AL progression in the control group between the first and second years (0.33 and 0.25 mm, respectively).^20^ Other previous studies have also shown the highest CARE value in the first year of treatment. For example, both Chamberlain et al., using dual‐focus CL (Misight, CooperVision, missight.com) and Walline et al. with multifocal +2.50 D addition CL (Biofinity, coopervision.com), reported that AL progression decreased in the experimental groups each year until the third year of the study, which could lead to greater efficacy with CL wear.^23^, ^29^ Using a meta‐analysis, Huang et al. stated that most interventions lose their early effect within the second year.^31^ However, after a review of the literature regarding axial elongation, Brennan et al. concluded that myopia progression over 3 years was highly correlated with the mean 1‐year progression and appeared to be approximately twice the 1‐year treatment effect.^32^ The present data indicated that the continued treatment effect was more than twice the 1‐year efficacy. The non‐significant decrease in CARE over time in the current study could be due to several factors, such as the inclusion of progressors, a wider range of ages and refractive errors, wearing SV‐spectacles rather than SV‐CL and the low number of dropouts given that children who experienced faster progression would be less motivated to continue. Another factor to consider could be the race of the participants. In a 2‐year follow‐up study of 74 Caucasian children, Ruiz‐Pomeda et al. did not find a reduction in the AL change during the second year in either the control or in the experimental group.^33^

The difference in SE after 36 months of follow‐up (0.74 D—Table 2) was similar to the value found by Chamberlain et al. (0.73 D) comparing MiSight and SV‐CL.^29^ Walline et al. observed a lower difference of 0.15 D and 0.47 D with Biofinity multifocal centre distance CL having +1.50 D and +2.50 D additions, respectively.^23^ Meanwhile, Ruiz‐Pomeda et al. reported a difference of 0.44 D between Misight and SV‐CL.^28^

The subjective impressions noted here were consistent with prior ratings, indicating that participants tolerated the soft myopia management CL well.^29^, ^40^, ^41^ In a recent systematic review and meta‐analysis, Ping et al. reported that subjective impression ratings for vision and comfort with myopia control CL were slightly lower than SV CL. Moreover, children rated their overall visual experience higher than young adults.^42^ The finding of just 31 mild corneal staining cases through the whole evaluation period, with no other adverse events, confirms the safety of the fitted CL. In agreement with the current study, Walline et al. also reported a low percentage of adverse events that did not cause discontinuation of lens wear.^23^ Further, Bullimore et al., in a review of seven prospective studies, reported the incidence of microbial keratitis in children wearing soft lenses to be no higher than found in adults, while the incidence of corneal infiltrative events seemed to be markedly lower.^43^

With respect to visual acuity, in agreement with the present study, Sankaridurg et al. found a reduction of half a line of logMAR binocular HCVA in Chinese children using the EDOF lens as compared with the control SV‐CL.^20^

As explained in the methodology, due to ethical and compliance reasons, it was considered appropriate to let participants choose their preferred optical strategy (CL or spectacles). Additionally, due to the nature of the trial, masking was not possible, but seemed unlikely to have affected the outcome given the high compliance rate. Nevertheless, the lack of randomisation is a limitation due to issues such as selection bias, confounding factors such as differences in the groups and limitations in the applicability of these results to wider populations. However, it is believed that this study is more representative of a real‐life scenario where patient motivation plays an important role in myopia management. For example, randomisation of children to test versus control groups may result in a greater number of discontinuations from the control group. A further limitation is that when the children in the spectacle group needed an updated refraction, they went to their eye care professional, and different standards for prescribing may have been adopted. Further, varying ophthalmic materials (e.g., polycarbonate, CR‐39 and high‐index lenses) have different optical properties, which could have introduced variability into the results. An additional limitation was that the data were compiled from a single centre.

In summary, in a Caucasian progressing paediatric population, 3 years of MYLO CL wear resulted in significantly slower myopia progression compared with a SV‐spectacle group. Additionally, CL wear provided good vision and was deemed safe over the 3‐year period in this group of lens wearers.

## AUTHOR CONTRIBUTIONS

**Sergio Díaz‐Gómez:** Conceptualization (equal); data curation (equal); formal analysis (equal); investigation (lead); writing – original draft (equal); writing – review and editing (equal). **Padmaja Sankaridurg:** Writing – original draft (equal); writing – review and editing (equal). **José A. López‐Garrido:** Data curation (equal); investigation (equal); writing – review and editing (supporting). **Mercedes Burgos‐Martínez:** Conceptualization (equal); writing – review and editing (supporting). **Jesús Carballo‐Álvarez:** Conceptualization (equal); data curation (equal); formal analysis (equal); methodology (equal); writing – original draft (lead); writing – review and editing (equal).

## FUNDING INFORMATION

Mark'ennovy manufactured and provided the contact lenses used in the study. Avizor provided the maintenance solutions.

## CONFLICT OF INTEREST STATEMENT

PS is an inventor on patents and patent application related to myopia control and is currently an employee of ZEISS Vision Care but has no financial or any other competing interests related to the subject matter of this article. M.B‐M. is an employee of mark'ennovy. This company has commercial interest in myopia control products. All the other authors report no financial interests or conflicts of interest.
